# Development of a Daily Living Self-Efficacy Scale for Older Adults in Japan

**DOI:** 10.3390/ijerph20043292

**Published:** 2023-02-13

**Authors:** Mizue Suzuki, Masahiro Shigeta, Takuya Kanamori, Marika Yokomichi, Masayo Uchiyama, Keigo Inagaki, Tomoyoshi Naito, Hajime Ooshiro, Yatami Asai

**Affiliations:** 1 School of Medicine, Hamamatsu University, 1-20-1 Handayama, Hamamatsu 431-3192, Japan; 2 School of Medicine, Jikei University, Tokyo 105-8461, Japan; 3 Totomi Hospital, Hamamatsu 434-0012, Japan; 4 Seirei Mikatahara General Hospital, Hamamatsu 433-8558, Japan

**Keywords:** older adults, self-efficacy, daily living

## Abstract

Objectives: Older adults tend to experience decreased enjoyment and fulfillment in life, social interactions, and independent living, with aging. These situations often result in lower levels of daily living self-efficacy in activities, which is one of the factors resulting in a decline in the quality of life (QOL) among older individuals. For this reason, interventions that help maintain daily living self-efficacy among older adults may also help maintain a good QOL. The objective of this study was to develop a daily living self-efficacy scale for the elderly that can be used to evaluate the effects of interventions aimed at enhancing self-efficacy. Methods: An expert meeting involving specialists in dementia treatment and care was held, to prepare a draft for a daily living self-efficacy scale. In the meeting, previous studies on self-efficacy among older adults, which were collected in advance, were reviewed, and the experiences of the specialists were discussed. Based on the reviews and discussions, a draft of a daily living self-efficacy scale comprising 35 items was prepared. This study on daily living self-efficacy was conducted from January 2021 to October 2021. The internal consistency and concept validity of the scale were evaluated based on the assessment data. Results: The mean age ± standard deviation of the 109 participants was 84.2 ± 7.3 years. The following five factors were extracted based on factor analysis: Factor 1, “Having peace of mind”; Factor 2, “Maintaining healthy routines and social roles”; Factor 3, “Taking personal care of oneself”; Factor 4, “Rising to the challenge”; and Factor 5, “Valuing enjoyment and relationships with others”. The Cronbach’s alpha coefficient exceeded 0.7, thereby suggesting sufficiently high internal consistency. Covariance structure analysis confirmed sufficiently high concept validity. Conclusions: The scale developed in this study was confirmed to be sufficiently reliable and valid, and when used during dementia treatment and care to assess the levels of daily living self-efficacy among older adults, it is expected to contribute to the improvement of QOL among older adults.

## 1. Introduction

Various mental and physical changes occur with aging, such as decreased cognitive function and an increased risk of frailty. As minor setbacks in daily life increase, older adults may become depressed more easily or even lose their zest for life. The scope and variety of their activities are narrowed down, possibly leading to declines in mental and physical function as a result of disuse. Specifically, impaired cognitive function among the elderly can result in feelings of despair and resignation, as well as lower levels of self-efficacy. Moreover, when older adults interact less with others, they tend to feel less certain about their existence and often become less motivated or self-confident [1].

Bandura defined self-efficacy as an individual’s belief in his or her capacity to execute necessary behaviors [2]. Based on Bandura’s theory of self-efficacy, Tinetti et al. developed the Fall Efficacy Scale (FES) to evaluate an individual’s confidence in his or her ability to avoid falls while performing activities, which included 10 items on the activities of daily living (ADL) and instrumental activities of daily living (IADL) [3]. Hill et al. developed a revised version of the FES comprising 14 items [4]. The FES is an evaluation index for the prediction of falls, and impaired mental and physical function that may occur in the future [5]. Referring to the FES, Suzuki et al. [6] developed a self-efficacy scale related to falls and attempted to evaluate self-efficacy in regard to ADL [7].

To maintain the quality of life (QOL) of older adults at a certain level, autonomy and well-being are important [8]. In addition, for older adults at risk of requiring nursing care, and for those suffering from dementia in particular, the maintenance of self-efficacy in daily living is considered essential for the maintenance of QOL. It is crucial for older adults to continue having confidence in their ability to conduct ADL. It was predicted that if older adults continue or regain confidence in their lives, the progression of dementia would be mitigated or prevented. The maintenance of self-efficacy can result not only in the maintenance of mental and physical functions, but also in the recovery of autonomy as well as the prolongation of life expectancy [9].

It is essential to maintain and enhance the life functions of older adults that improve their levels of daily living self-efficacy, such as enjoyment and fulfillment in life, social interactions, and independent living. However, to the best of our knowledge, no scale has been developed for measuring daily living self-efficacy levels among older adults in general. If daily living self-efficacy among older adults in general can be measured, the impact of dementia treatment and care on the levels of self-efficacy among older adults can be evaluated. Medical treatment and care approaches that can enhance self-efficacy levels among older adults can also improve their QOL. Therefore, this study aimed to develop a daily living self-efficacy scale for older adults, including those at risk of requiring nursing care, and to verify its reliability and validity.

## 2. Materials and Methods

### 2.1. Development of a Daily Living Self-Efficacy Scale for Older Adults

From September 2020 to November 2020, the authors performed a review of the literature on studies on existing scales related to self-efficacy among older adults and patients with chronic diseases [6,7], as well as self-efficacy in general. Daily living was defined as the enjoyment of and fulfillment in life, social interactions, forgetfulness, activities, and independence in daily living among older adults. Based on the results, items that could potentially be included in a self-efficacy scale were extracted and rephrased into words and expressions that were easier for older adults to understand.

In this study, self-efficacy was defined in accordance with Bandura’s definition, as “the level of an individual’s belief in his or her capacity to execute necessary behaviors well”, to prepare the draft of the daily living self-efficacy scale in general [2]. As interventions intended for the recovery of self-confidence in and motivation for daily living are being provided in medical practice and in the care of older adults, measuring the effects of these interventions is expected to be one of the main uses of the scale.

An expert meeting involving physicians, mental health social workers, certified dementia nurses, and certified geriatric nurse specialists was held. The items that constitute the daily living self-efficacy scale and the adequacy of expressions in the scale were discussed, and a pilot version comprising 35 items was prepared. Using the scale involved having the participants respond to each question in regard to how confident they were for each item by pointing their finger at one of the four choices, from “4: Very confident” to “1: Not confident”. The examiner read the question and answer choices aloud, while showing the participants the sheet with the choices displayed.

Prior to the data collection process, 10 older persons participating in a care prevention project were tested using the pilot version of the scale to assess whether they could answer each question without difficulty. Older patients with Alzheimer-type dementia, older patients with mild cognitive impairment, and patients with early-onset dementia (*n* = 3) were included, so that the scale developed in this study could be used for older patients with impaired cognitive function. Impressions or comments regarding the ease of answering each question were collected from those who were tested, and the items of the scale were reorganized accordingly, thereby resulting in a total of 31 items.

### 2.2. Selection Criteria of Participants

In addition to the attendants of care prevention classes, older individuals in need of nursing care attending day-care services were included as participants, because the scale being developed was intended not only for those at risk of requiring nursing care, i.e., those with frailty, but also those currently in need of nursing care. Only the individuals who provided consent to participation in the study were examined. The selection criteria included older adults who could respond to the interview questions in the survey, those who attended day-service or care prevention classes, and those who provided consent to participate in the study.

### 2.3. Study Period and Procedures

In this study, the data were collected, by nurses with at least five years of experience in geriatric nursing, from January 2021 to October 2021. The participants involved in this study were older adults with a Mini-Mental State Examination (MMSE) score of 13 points or higher, and who had answered at least two of the three preliminary questions correctly. Referring to the development of the Japanese version of the dementia quality of life instrument (DQOL) [10], the preliminary questions were set to determine in advance whether the participants could understand the questions in this survey and select answers from the choices presented. Individuals meeting these criteria were included in the survey regardless of whether they had been diagnosed with dementia. In this study, the participants included older adults with dementia and older adults who were still able to respond appropriately to the questionnaire items, even though their cognitive function was declining. This is because it is important to measure and improve the daily living self-efficacy of older adults diagnosed with dementia or who are beginning to experience cognitive decline.

In addition to the self-efficacy scale for older adults, the MMSE and a subjective QOL scale were used. Data on the attributes of the participants were obtained from the attendance records of care prevention classes or day-care services. The evaluation of their daily activities and an assessment using the Gottfries–Brane–Steen scale (GBS) were performed based on the information obtained from the staff in charge.

### 2.4. Evaluation

#### Characteristics of the Participants

Data on the participants’ gender, age, underlying conditions for dementia, and physical complications, were obtained from the above-mentioned attendance records.

### 2.5. Evaluation of Concurrent Validity

#### 2.5.1. ADL (Katz)

A three-point evaluation index (“independent” [1 point], “partially assisted” [2 points], and “fully assisted” [3 points]) was used for the measurement of basic ADL, namely, independence when bathing, dressing, toileting, transferring, continence, and feeding [11]. Higher scores indicated lower levels of ADL.

#### 2.5.2. Mini-Mental State Examination (MMSE)

The MMSE is a screening examination for cognitive function. It comprises the following subscales: orientation in time (5 points) and place (5 points), registration (3 points), attention and calculation (5 points), recall (3 points), language (8 points), and copying (1 point) [12]. In this study, the total MMSE score was used for the statistical analysis. Lower scores indicated decreased cognitive function.

#### 2.5.3. Gottfries–Brane–Steen Scale (GBS)

The GBS is a comprehensive dementia rating scale, and it comprises four sub-scales, as follows: “A. Motor function” (six items, 0–36 points), “B. Intellectual function” (eleven items, 0–66 points), “C. Emotional function” (three items, 0–18 points), and “D. Psychiatric symptoms” (six items, 0–36 points). Lower scores indicated better conditions [13,14].

#### 2.5.4. Subjective QOL Scale for Older Patients with Dementia 

The dementia quality of life instrument (DQOL), which is a subjective QOL scale specific to older patients with dementia developed by Brod et al. [15], comprises five subscales, as follows: “Self-esteem”, “Positive affect/humor”, “Negative affect”, “Feelings of belonging”, and “Sense of aesthetics”. The Japanese version has been evaluated in terms of reliability and validity [10,15], and can be used for the measurement of subjective QOL. For example, “Positive affect/humor” represents positive emotions, including humor, having fun, and being full of energy; “Sense of aesthetics” represents the sense of being conscious about and enjoying music, animals, or nature, among others.

### 2.6. Ethical Considerations

The outline for the study was described in the questionnaire. The survey was conducted anonymously to avoid personal identification. The protection of privacy and the presentation of study data at academic conferences were explained in writing. The individuals who provided written consent were included in the study. This study was approved by the Ethics Committee of Hamamatsu University School of Medicine (No. 20-284). For the participation of an individual certified as requiring nursing care, consent was obtained from a family member, as well as the individual him/herself.

### 2.7. Statistical Analysis

The participants that provided missing values in the survey items were excluded from the data. Mean values and standard deviations (SDs) were calculated for each item. Item–total (IT) correlation analyses were performed, and the correlation coefficient of the total score and each item was confirmed. An exploratory factor analysis (principal factor method with varimax rotation) was also performed, and the items with high factor loading for two factors were removed to determine the factors. After determining the factors, with regard to reliability, the Cronbach’s alpha coefficient was calculated to assess internal consistency. For the concordance rate, the Pearson correlation coefficient was used. For construct validity, a covariance structure model was tested for goodness of fit (GFI). For validity in terms of the relationship with other scales, correlation coefficients with ADL, MMSE, GBS, and the Japanese version of the DQOL were calculated. IBM SPSS statistics and AMOS version 21 (IBM, Chicago, IL, USA) were used for all statistical analyses.

## 3. Results

### 3.1. Participants’ Characteristics

The total number of participants involved in this study included 185 older adults participating in two-day services and two care prevention classes. Of the 149 older adults (80.5%) who provided consent, 127 (68.64%) met the inclusion criteria, which were an MMSE score of 13 or higher, and correct answers to two of the three preliminary questions. The participants that provided missing data were excluded from the data analysis, and as a result, the final number of participants was 109 (58.9%) older adults. Twenty-five participants (22.9%) were male and 84 (77.1%) were female (Table 1). The mean age of the participants was 84-years old (Mean 84.2; SD = 7.3). Sixty-one participants (56.0%) were involved in care prevention programs, and 48 (44.0%) were users of day-care services. Based on family configuration, the largest number of participants (55 participants, 50.5%) lived with their children, followed by those who lived alone (23 participants, 21.1%). The nursing care levels determined by the public long-term care system in Japan (from 1 to 5, based on an assessment of the care requirement) was “independent” for 55 participants (50.5%) and “long-term care level-1” for 25 participants (22.9%). Thirteen participants (11.9%) had been diagnosed with dementia. The most prevalent type of dementia was Alzheimer-type (five participants, 38.5%). The most frequently observed past or current disease was motor function disorder (44 participants, 40.4%), followed by circulatory disorder (35 participants, 32.1%). The most common physical function disorder was gait disturbance (46 participants, 42.2%), followed by hearing impairment (38 participants, 34.9%).

The mean values of each scale are listed in Table 2. The mean ± SD ADL (Katz) and MMSE values were 6.8 ± 2.1 and 25.2 ± 4.5, respectively. The GBS item with the highest mean score was “B: Intellectual impairment” (3.98 ± 8.63), and the DQOL item with the highest mean score was “Negative affect” (43.3 ± 6.4).

### 3.2. Mean Values of and Reliability Analysis of IT Correlation Coefficients and Test–Retest Reliability

The mean values and IT correlation coefficients of the items included in the daily living self-efficacy scale are listed in Table 3. Among these items, the mean value for “Having someone to rely on during an emergency” was the highest (3.76), followed by that for “Taking daily medications” (3.73). All the IT correlations were significant, ranging from 0.300 to 0.650. The test–retest reliability of each item after one week generated a score of over 0.827.

### 3.3. Verification of Reliability Using Cronbach’s Alpha and Test–Retest Reliability

Firstly, exploratory analysis using the Shapiro–Wilk test of normality was used to confirm the normal distribution of data, after which factor analysis was conducted. The data obtained from exploratory factor analysis, including the results of the factor analysis and the Cronbach’s alpha for the daily living self-efficacy scale for older adults are listed in Table 3. The test–retest reliability after one week generated a score of 0.927.

### 3.4. Verification of Construct Validity on Factor Analysis as Exploratory Analysis

Based on the results of the factor analysis, the items with a factor loading below 0.4 and those with high factor loading for two factors were deleted, and the following five factors involving 23 items were selected: Factor 1, “Having peace of mind”; Factor 2, “Maintaining healthy routines and social roles”; Factor 3, “Taking personal care of oneself”; Factor 4, “Rising to the challenge”; and Factor 5, “Valuing enjoyment and relationships with others”. The Cronbach’s alpha was 0.72.

The final version comprises 23 items. The eight items deleted from the initial 31 items include: living peacefully every day, having a purpose in life, talking to people by oneself, talking to people who have trouble communicating with me until they understand me, helping people in need, asking for help when in trouble, going to the bathroom, and taking a bath (Appendix A).

### 3.5. Verification of Construct Validity and Covariance Structure Analysis

**Figure 1:**
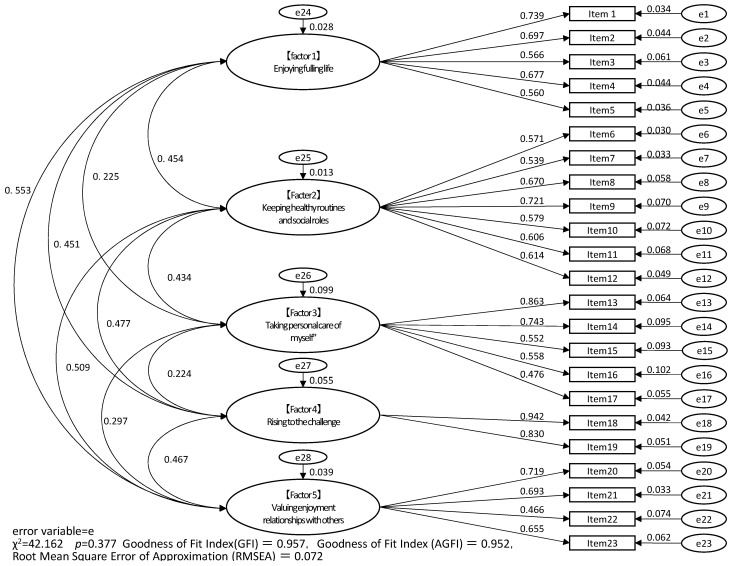
Results of confirmatory factor analysis of the daily living self-efficacy scale for older adults.

The results of the covariance structure analysis, which was performed as a confirmatory factor analysis to evaluate concept validity, are shown in Figure 1. The five factors extracted in the exploratory factor analysis were used as latent variables. A model assuming covariance among the latent variables was set up, and covariance structure analysis was conducted. The model with the best fit was selected. In Figure 1, e1 to e28 indicate the error variables that are not reflected in the model.

All standardized coefficients were significant. The daily living self-efficacy scale model comprised five factors (χ^2^ = 42.162, *p* = 0.377). The GFI indices were as follows: GFI = 0.957, adjusted GFI = 0.952, and the root-mean-square error of approximation = 0.072. Thus, the GFI was statistically verified. The path coefficients between the latent and observed variables were significant and ranged from 0.466 to 0.942.

### 3.6. Verification of Concurrent Validity on the DQOL of “Positive Affect/Humor”

The correlation coefficients between the self-efficacy scale for older adults or age, and other scales, are listed in Table 4.

The Japanese version of the DQOL, “Positive affect/humor” showed significant positive correlations with all five factors of the self-efficacy scale. “Self-esteem” showed significant positive correlations with three factors: Factors 1, 2, and 5 (correlation coefficients; 0.210–0.396).

### 3.7. Correlation Coefficients of Age, ADL, and MMSE

No significant correlation was found between age and each factor of the self-efficacy scale. The Katz ADL showed significant negative correlations with Factor 2, “Maintaining healthy routines and social roles” (correlation coefficient, −0.202) and Factor 3, “Taking personal care of oneself” (correlation coefficient, −0.356). The MMSE showed significant positive correlations with Factor 2, “Maintaining healthy routines and social roles” (correlation coefficient, 0.243) and Factor 3, “Taking personal care of oneself” (correlation coefficient, 0.308).

### 3.8. Verification of Convergent Validity on the DQOL of “Sense of Aesthetics”

Regarding the Japanese version of the DQOL, “Sense of aesthetics” also showed significant positive correlations with all five factors of the self-efficacy scale. “Self-esteem” showed significant positive correlations with three factors: Factors 1, 2, and 5 (correlation coefficients: 0.210–0.396).

### 3.9. Verification of Discriminant Validity

Among the items of the GBS, “A: Motor function” showed significant negative correlations with three factors, from Factor 2 to Factor 5 of the daily living self-efficacy scale (correlation coefficients, −0.193 to −0.352), and was not significantly correlated (*p* = 0.059), or negatively correlated with Factor 1 of the daily living self-efficacy scale.

## 4. Discussion

In this study, we developed a scale for measuring daily living self-efficacy among older adults. The reliability and validity of the scale were assessed among older adults at risk of requiring nursing care (participants of care prevention projects) and those currently receiving nursing care (covered by nursing care insurance or certified as requiring nursing care).

### 4.1. Participants’ Characteristics

The cognitive functions of the participants were assessed using the MMSE, and the mean score was 25.2 points. The levels of ADL were assessed using the Katz ADL scale, and the mean score was 6.8 points. These results indicate that many participants were independent when performing ADL. The level of necessity of nursing care was “independent” among approximately half of the participants, and among most of those requiring nursing care, the level of necessity was low (“Nursing care level 1”). A few participants requiring high levels of care (“Nursing care level 4” and “Nursing care level 5”) had severe physical dysfunctions, such as the sequelae of stroke, but they were eligible to participate in the survey.

### 4.2. Deleted Items in the Daily Living Self-Efficacy Scale for Older Adults

Items in the self-efficacy scale for older adults were carefully discussed between the co-researchers (gerontology specialists), based on eight items with low factor loadings in the factor analysis. Based on these discussions, eight items were deleted, resulting in 23. The following items were difficult to understand and too challenging for the participants and were, therefore, removed: “living peacefully every day”, “having a purpose in life”, “talking to people by oneself”, “talking to people who have trouble communicating with me until they understand me”, “helping people in need”, and “asking for help when in trouble”. Furthermore, the items “going to the bathroom” and “taking a bath” were deleted because these items focused on ADL performance.

The deletion of items was reviewed by the experts involved in the development of the scale, and it was concluded that the content of the scale was further refined as a measurement of daily living self-efficacy.

### 4.3. Construct Validity Exploratory Factor Analysis

The factor analysis of the data collected using the daily living self-efficacy scale developed in this study demonstrated a five-factor structure comprising the following factors: Factor 1, “Having peace of mind”, Factor 2, “Maintaining healthy routines and social roles”, Factor 3, “Taking personal care of oneself”, Factor 4, “Rising to the challenge”, and Factor 5, “Valuing enjoyment and relationships with others”.

Factor 1, “Having peace of mind”, comprised the following items: “Enjoying conversations with friends and family”, “Enjoying spending time with friends and family”, and “Having someone to rely on during an emergency”, which indicate relationships with people the participants can trust, and “Having a good life” as well as “Feeling fulfilled every day”, which indicate a positive evaluation of the self, with five items in total. Lawton pointed out the importance of QOL and psychological well-being among older adults [16], and developed positive affect (PA), which can be used for the evaluation of enjoyable activities and interactions with others [17]. Lawton also reported that PA affected QOL. Lawton’s study suggested the adequacy of the fact that the five items included in “Having peace of mind” were established as Factor 1 in this study.

Factor 2, “Maintaining healthy routines and social roles”, included the following items: “Going out at least once a week” and “Taking daily medications”, which indicate daily routines, as well as “Helping others”. Older adults are often recipients of nursing care, with their family members being the providers. However, among older adults, their engagement in activities that help others may result in the enhanced maintenance of equal relationships with others and possibly, self-efficacy.

Rabins and Kasper developed Alzheimer’s Disease-Related Quality of Life (AD-QOL) as a health-related QOL scale specific to dementia, and they included “social interaction” and “relationship with surroundings” as QOL domains [18]. Maintaining certain relationships with others can result in the maintenance of social roles. Additionally, items related to the autonomy of older adults, such as “Accomplishing important tasks to the end” would be associated with social roles.

As for Factor 3, “Taking personal care of oneself” represents self-confidence in “Buying daily necessities” and “Withdrawing money from banks and post offices”. Older adults are highly likely to experience interferences with daily living because of age-related physical and mental changes.

Suzuki et al. attempted to measure self-efficacy in ADL, and they reported that the maintenance of functions for ADL, including IADL, was critical for the maintenance of self-efficacy among older adults living in local communities [6]. Factor 4, “Rising to the challenge”, comprised two items: “Snapping out of it when depressed” and “Remaining positive despite failure”, which are closely related to self-efficacy among older adults. Factor 5, “Valuing enjoyment and relationships with others”, included various items, such as “Doing what I love and having fun” and “Being energetic and feeling good”. Yamamoto-Mitani et al. raised control of emotion as a subscale of the QOL scale for older patients suffering from dementia [19], and Perach et al. pointed out that the control of emotions is crucial in decision making among older patients suffering from dementia [20]. These findings suggest that among older adults with declining cognitive functions, it is crucial to maintain a good emotional status by valuing enjoyment and relationships with others to ensure the maintenance of self-efficacy.

An examination of the construct validity of the scale proposed in this study showed that both Factor 2, “Maintaining healthy routines and social roles”, and Factor 3, “Taking personal care of oneself”, were significantly associated with ADL and MMSE. Conn pointed out that a decline in physical function with increased age is a factor influencing decreased levels of self-efficacy [21]. Similar results were obtained in this study with regard to the impact of physical and cognitive functions. Moreover, because Factor 2 includes an item related to social roles, and Factor 3 includes an item on self-confidence in IADL, these factors may influence judgment in social life, IADL, the characteristics of actions, and the characteristics of behaviors, such as cognitive function.

### 4.4. Reliability of Cronbach’s Alpha and Test–Retest

Regarding reliability, Cronbach’s alpha values showed scores of 0.7 or higher for individual factors, thereby indicating sufficient internal consistency. Concerning the confirmatory factor analysis, the criteria for the GFI indices were met, thereby confirming the validity of the factor structure involving five factors and 23 items. Although the participants involved in this study included 13 older adults who were diagnosed with dementia, the test–retest reliability after one week generated a score of 0.927, and the reliability of the scale was obtained satisfactorily.

### 4.5. Concurrent and Convergent Validity

All the subscale items of the Japanese version of the DQOL, “Positive affect/humor” were significantly associated with each factor of the self-efficacy scale.

### 4.6. Concurrent Validity

In this study, daily living self-efficacy is linked to feelings of “Positive affect/humor” as a test for concurrent validity. Self-efficacy in ADL result in feelings of self-affirmation. In this study, “Positive affect/humor” was used to vivificate concurrent validity. All the subscale items in the Japanese version of the DQOL “Sense of aesthetics” were significantly associated with each factor of the self-efficacy scale. Aesthetics is a component of the QOL that supports daily living self-efficacy, and it was used for the purpose of convergent validity. These items involving positive affect and sense of aesthetics are related to the emotional states raised by Bandura and Cervone [22] as antecedent factors for self-efficacy. Their significant associations with all the items contained in the self-efficacy scale clearly indicates the validity of the self-efficacy scale proposed in this study.

Moreover, a significant association was found between the subscale of the Japanese version of the DQOL “Feelings of belonging”, and Factor 5, “Valuing enjoyment and relationships with others”, thereby further suggesting that the feeling of belonging that motivates older adults to help others, or that they experience when they feel loved by others, is one of the factors supporting self-efficacy.

Based on the results mentioned above, the reliability and validity of the self-efficacy scale for older adults were confirmed. This study is expected to be useful for the evaluation of the effects of care interventions intended for the enhancement of self-efficacy among older adults, as well as the accumulation of evidence for the evaluation of relationships between self-efficacy and cognitive/physical functions among the elderly for the purpose of extending life expectancy.

### 4.7. Limitations and Directions for Future Research

This study involved 109 participants. According to Floyd, for a sample size of 100 participants, five participants per variable would be considered adequate to yield reliable results, whereas 10 participants per variable would be sufficient for a sample size of less than 100 participants [23]. Therefore, a sample size of 138 participants in this study was not sufficient for factor analysis of the exploratory analysis and covariance structure analysis.

This study did not obtain enough participants owing to the pandemic caused by the coronavirus disease 2019 (COVID-19). Therefore, future studies on the subject of this study must consider enhanced factor analysis.

This study was developed to support older adults with MMSE scores of 13 or higher, participating in day services and care prevention classes, to maintain daily living self-efficacy, even in the face of cognitive decline. Therefore, the participants involved in this study were older adults with mild cognitive impairments, and this aspect may have resulted in biased results.

In order to ensure the reliability of the responses, only those participants who were able to respond appropriately were included in this study. Therefore, participants who were suffering from cognitive decline and could not respond appropriately were removed from the study. This is the limitation of the study, and we plan to work on ways to further assess older adults with cognitive decline in regard to self-efficacy in the future.

This study involved older individuals attending care prevention classes and day-care services. These participants do not represent the general older population. In the future, to expand the use of the daily living self-efficacy scale proposed in this study, we plan to test this scale among healthy older adults or patients suffering from advanced dementia. Through this scale, we expect to clarify the impact of the treatment and care of older patients suffering from dementia on both self-efficacy and life expectancy.

## 5. Conclusions

The self-efficacy scale developed in this study was confirmed to be partially reliable and valid. The number of participants involved in this study was small, and the sample was limited. As a result, the further verification of reliability and validity is required in associated future studies. This study involved a small number of participants and limited sampling of day services and care prevention classes, and thus, the further verification of reliability and validity is required in future studies.

When used to conduct dementia treatment and care to assess the levels of daily living self-efficacy among older adults, the daily living self-efficacy scale proposed in this study is expected to contribute to the improvement of QOL among older patients suffering from dementia.

## Figures and Tables

**Table 1:** Participants’ attributes.

Item			N	%
Sex	Male		25	22.9
	Female		84	77.1
	Total		109	100.0
Family structure	Living with child(ren)		55	50.5
	Living alone		23	21.1
	Living with spouse		15	13.8
	Living with spouse and child(ren)		14	12.8
	Living in a facility		1	0.9
	Living with grandchild(ren)		1	0.9
	Total		109	100.0
Affiliation	Care prevention class		61	56.0
	Day-care service		48	44.0
	Total		109	100.0
Nursing care level	Independent		55	50.5
	Support level 1		5	4.6
	Support level 2		6	5.5
	Nursing care level 1		25	22.9
	Nursing care level 2		9	8.3
	Nursing care level 3		6	5.5
	Nursing care level 4		1	0.9
	Nursing care level 5		2	1.8
	Total		109	100.0
Type of dementia diagnosis	Alzheimer-type dementia		5	4.6
	Vascular dementia		1	0.9
	Mild cognitive impairment		1	0.9
	Sequela of encephalitis		1	0.9
	Unknown type of dementia No diagnosis of dementia		5 96	4.6 88.1
	Total		109	100.0
Past/current disease	Motor function disorders			
		Yes	44	40.4
		No	65	59.6
	Total		109	100.0
	Circulatory disorders			
		Yes	35	32.1
		No	74	67.9
	Total		109	100.0
	Cerebrovascular disorders			
		Yes	27	24.8
		No	82	75.2
	Total		109	100.0
	Gastrointestinal disorders			
		Yes	17	15.6
		No	92	84.4
	Total		109	100.0
	Respiratory disorders			
		Yes	11	10.1
		No	98	89.9
	Total		109	100.0
	Renal/urological disorders			
		Yes	4	3.7
		No	105	96.3
	Total		109	100.0
Physical function disorder	Gait disturbance			
		Yes	46	42.2
		No	63	57.8
	Total		109	100.0
	Hearing impairment			
		Yes	38	34.9
		No	71	65.1
	Total		109	100.0
	Visual impairment			
		Yes	22	20.2
		No	87	79.8
	Total		109	100.0
	Contracture			
		Yes	22	20.2
		No	87	79.8
	Total		109	100.0
	Paralysis			
		Yes	21	19.3
		No	88	80.7
	Total		109	100.0

**Table 2:** Mean value of each scale.

Scale	Mean	SD	Minimum Value	Maximum Value
ADL (Katz) (6–18)	6.81	2.07	6	17
Mini-Mental State Examination (MMSE)	25.17	4.47	10	30
Gottfries–Brane–Steen scale (GBS)				
A: Motor function	2.36	5.67	0	36
B: Intellectual function	3.98	8.63	0	48
C: Emotional reaction	0.62	1.65	0	11
D: Psychiatric symptoms	0.83	2.43	0	16
Japanese version of the dementia quality of life instrument (DQOL)				
Self-esteem	14.29	3.15	6	20
Positive affect/humor	20.81	4.32	8	28
Negative affect	43.28	6.36	29	55
Feelings of belonging	9.75	2.50	3	15
Sense of aesthetics	17.00	4.33	5	25

**Table 3:** Factor analysis of the daily living self-efficacy scale for older adults. * *p* < 0.001.

Item	Item	Factor 1	Factor 2	Factor 3	Factor 4	Factor 5	Cronbach’s Alpha	Mean	SD	IT Correlation Coefficient
Factor 1: Having peace of mind							0.789			
1	Enjoying conversation with friends and family	0.733	0.28	0.127	0.289	−0.182		3.50	0.65	0.588 *
2	Enjoying spending time with friends and family	0.645	0.049	0.173	0.34	0.008		3.50	0.68	0.553 *
3	Having a good life	0.599	0.005	0.051	−0.009	0.223		3.17	0.78	0.420 *
4	Feeling fulfilled every day	0.567	0.202	0.077	0.047	0.302		3.35	0.71	0.560 *
5	Having someone to rely on during an emergency	0.466	0.389	−0.059	−0.046	0.093		3.76	0.59	0.441 *
Factor 2: “Maintaining healthy routines and social roles”							0.804			
6	Going out at least once a week	0.171	0.677	−0.042	−0.012	0.117		3.70	0.55	0.438 *
7	Taking daily medications	0.174	0.666	0.111	0.062	−0.148		3.73	0.56	0.470 *
8	Accomplishing important tasks to the end	−0.006	0.561	0.256	0.32	0.154		3.33	0.82	0.595 *
9	Doing what I want to do	0.313	0.546	0.17	0.137	0.19		3.28	0.93	0.595 *
10	Devising ways to avoid failure due to forgetfulness	0	0.456	0.269	0.225	0.143		3.18	0.86	0.541 *
11	Helping others	0.062	0.434	0.347	0.218	0.379		3.00	0.85	0.650 *
12	Making decisions for myself	0.309	0.419	0.358	0.142	0.045		3.53	0.73	0.621 *
Factor 3: “Taking personal care of oneself”							0.780			
13	Buying daily necessities	0.108	0.132	0.852	0.014	0.123		3.32	0.91	0.612 *
14	Withdrawing money at banks and post offices	0.065	0.127	0.690	−0.006	0.103		3.22	1.07	0.525 *
15	Communicating by phone	0.003	−0.047	0.591	0.209	−0.11		3.45	0.96	0.376 *
16	Preparing simple meals	0.054	0.127	0.554	0.045	0.006		3.16	1.01	0.460 *
17	Choosing clothes according to season and occasion	0.111	0.073	0.483	−0.019	0.117		3.61	0.71	0.429 *
Factor 4: “Rising to the challenge″							0.869			
18	Snapping out of it when depressed	0.171	0.316	0.075	0.785	0.274		3.36	0.67	0.625 *
19	Staying positive despite failure	0.247	0.126	0.077	0.748	0.243		3.26	0.77	0.564 *
Factor 5: Valuing enjoyment and relationships with others							0.721			
20	Doing what I love and having fun	0.427	0.188	0.247	0.061	0.461		3.21	0.79	0.626 *
21	Being energetic and feeling good	0.443	0.197	0.142	0.139	0.457		3.21	0.61	0.594 *
22	Memorizing people’s names and faces	0.064	−0.012	0.002	0.19	0.456		2.53	0.79	0.300 *
23	Being trusted by others	0.349	0.326	0.099	0.16	0.421		2.85	0.82	0.610 *
	Eigenvalue	8.647	2.886	1.887	1.84	1.584				
	Proportion of variance explained	27.893	10.550	7.672	6.801	5.609				
	Cumulative proportion of variance explained	27.893	38.443	46.115	52.916	58.525				
	Cronbach’s alpha	0.881								
	Kaiser–Meyer–Olkin measure of sample adequacy		0.792	*p* < 0.000						* *p* < 0.001
	Principal factor method, varimax rotation									

**Table 4:** Correlation coefficients between the daily living self-efficacy scale for older adults and age, and other scales.

Item	Correlation Coefficient	Factor 1	Factor 2	Factor 3	Factor 4	Factor 5
		Enjoying a Fulfilling Life	Maintaining Healthy Routines and Social Roles	Taking Personal Care of Oneself	Rising to the Challenge	Valuing Enjoyment and Relationships with Others
Age	r	0.115	−0.073	−0.116	−0.052	0.082
	*p* value	0.235	0.460	0.229	0.591	0.401
ADL (Katz)	r	−0.101	−0.202	−0.356	−0.149	−0.177
	*p* value	0.298	0.038	0.000	0.124	0.067
Mini-Mental State Examination (MMSE)	r	0.002	0.243	0.308	0.113	−0.001
	*p* value	0.980	0.015	0.002	0.255	0.991
Gottfries–Brane–Steen scale (GBS)						
A: Motor function	r	−0.181	−0.266	−0.352	−0.225	−0.193
	*p* value	0.059	0.006	0.000	0.019	0.046
B: Intellectual function	r	−0.096	−0.23	−0.147	0.021	−0.05
	*p* value	0.319	0.018	0.128	0.832	0.605
C: Emotional function	r	−0.185	−0.228	−0.200	−0.028	−0.121
	*p* value	0.054	0.019	0.037	0.771	0.211
D: Psychiatric symptoms	r	−0.073	−0.255	−0.053	0.057	−0.052
	*p* value	0.450	0.008	0.586	0.559	0.590
Japanese version of the dementia quality of life instrument (DQOL)						
Self-esteem	r	0.210	0.396	0.154	0.160	0.268
	*p* value	0.029	0.000	0.112	0.100	0.005
Positive affect/humor	r	0.504	0.297	0.198	0.326	0.444
	*p* value	0.000	0.002	0.040	0.001	0.000
Negative affect	r	0.168	0.041	−0.006	0.174	0.165
	*p* value	0.082	0.676	0.948	0.073	0.089
Feeling of belonging	r	0.093	0.099	−0.006	0.081	0.307
	*p* value	0.345	0.318	0.949	0.409	0.001
Sense of aesthetics	r	0.372	0.337	0.324	0.289	0.368
	*p* value	0.000	0.000	0.001	0.002	0.000

## Data Availability

The data presented in this study are available on request from the corresponding author. The data are not publicly available due to contains personal information.

## Appendix A

Please point to one item in the response sheet in the interview survey and show how confident you are in your ability to currently carry out the items I am about to show you. Please make sure your choices are based on the following options: ‘very confident’ to ‘not confident’.

**Table A1:** Questionnaire and Response Sheet for the Daily Living Self-Efficacy Scale for Older Adults.

	Item	Not Confident	Not So Confident	Fairly Confident	Very Confident
1	Enjoying conversation with friends and family	1	2	3	4
2	Enjoying spending time with friends and family	1	2	3	4
3	Having a good life	1	2	3	4
4	Feeling fulfilled every day	1	2	3	4
5	Having someone to rely on in during emergency	1	2	3	4
6	Going out at least once a week	1	2	3	4
7	Taking daily medications	1	2	3	4
8	Accomplishing important tasks to the end	1	2	3	4
9	Doing what I want to do	1	2	3	4
10	Devising ways to avoid failure due to forgetfulness	1	2	3	4
11	Helping others	1	2	3	4
12	Making decisions for myself	1	2	3	4
13	Buying daily necessities	1	2	3	4
14	Withdrawing money at banks and post offices	1	2	3	4
15	Communicating by phone	1	2	3	4
16	Preparing simple meals	1	2	3	4
17	Choosing clothes according to season and occasion	1	2	3	4
18	Snapping out of it when depressed	1	2	3	4
19	Staying positive despite failure	1	2	3	4
20	Doing what I love and having fun	1	2	3	4
21	Being energetic and feeling good	1	2	3	4
22	Memorizing people’s names and faces	1	2	3	4
23	Being trusted by others	1	2	3	4
